# Lumbar Facet Joint Cyst Treated With Decompression and Interlaminar Stabilization

**DOI:** 10.7759/cureus.9391

**Published:** 2020-07-25

**Authors:** Navraj S Sagoo, Harvinder Bhatti, Scott E Rowe, Ishaan Sharma, Ali S Haider

**Affiliations:** 1 Orthopedic Surgery, The University of Texas Medical Branch at Galveston, Galveston, USA; 2 Orthopedic Surgery, Atlanta Spine Surgery Center, Atlanta, USA; 3 Neurosurgery, University of Texas Southwestern, Dallas, USA; 4 Neurosurgery, Nova Southeastern University College of Osteopathic Medicine, Fort Lauderdale, USA; 5 Orthopedic Surgery, Ross University School of Medicine, Bridgetown, USA; 6 Neurosurgery, Texas A&M University College of Medicine, Houston, USA; 7 Neurosurgery, Baylor Scott & White Medical Center, College Station, USA

**Keywords:** facet joint cyst, coflex, decompression

## Abstract

Spinal facet joint cysts (FJCs) are an increasingly reported cause of lower back pain, radiculopathy, and neurological deficits owing to their proximity to adjacent nerve roots. The etiology of these cysts has not yet been fully elucidated, although they appear to be related to degenerative changes in the facet joints themselves. Conservative management strategies including physical therapy and oral analgesics rarely result in spontaneous regression of an FJC, often providing only short-term relief. The current treatment modalities reported for FJCs generally range from percutaneous cyst aspiration to decompression surgery with or without instrumented spinal fusion. However, instrumented fusion often results in a higher rate of complications such as pseudoarthrosis and adjacent segment disease. The Coflex^®^ (Paradigm Spine, LLC, New York, NY) interlaminar stabilization (ILS) implant is a novel titanium, U-shaped device which acts as a motion-preserving stabilizer and has proven to be a viable alternative to instrumental fusion. Here, we discuss a case of an FJC treated with both decompression and placement of a Coflex ILS device.

## Introduction

Spinal facet joint cysts (FJCs) are rare, benign lesions that arise from a defect within the facet joint capsule, with or without communications with the joint itself [1,2]. Most FJCs develop within the lumbar region, and often in sites of degenerative changes or instability [3,4]. Accordingly, FJCs are frequently noted as a condition of an aging population with a mean age in the sixties [5]. Although often asymptomatic and found incidentally, FJCs may rarely compress adjacent neural structures resulting in lumbar radiculopathy or neurological deficits [6,7]. The treatment modalities reported for FJCs generally range from percutaneous cyst aspiration to decompression surgery with or without instrumented spinal fusion [5].

Instrumented fusion eliminates fixed segment motion, often resulting in a higher rate of complications such as pseudoarthrosis and adjacent segment disease [8]. As an alternative to rigid fusion, the Coflex® (Paradigm Spine, LLC, New York, NY) interlaminar stabilization (ILS) device is a novel, motion-preserving technique that has shown comparable efficacy in patients with lumbar stenosis [8,9]. Here, we discuss a unique case of an FJC treated with both decompression and placement of a Coflex ILS device.

## Case presentation

A 60-year-old male patient presented to our institution with a six-month history of pain in the right buttock with radiation to the lateral aspect of the right thigh down to the right lateral calf and lateral foot. He had severe, unremitting pain associated with numbness and paresthesias. His pain progressively worsened despite conventional measures of non-operative management including physical therapy, anti-inflammatories, activity modification, muscle relaxers, narcotics, chiropractic manipulations, and multiple injections via pain management which included aspiration providing one day of relief. The patient reported no history of chronic inflammatory disease or prior traumatic events. The pain was aggravated when standing or walking and alleviated when lying down. He denied any bowel or bladder dysfunction. The visual analog scale for assessing pain reached 5 out of 10.

On physical examination, his gait was antalgic with no obvious pelvic obliquity. Flexion and extension of the lumbar spine caused severe pain, with extension more than flexion. Straight leg raise was concordant with a dermatomal radicular pain into the right L5/S1 distribution. Femoral stretch test was negative. Muscle strength was normal with no motor deficits. Achilles and quadriceps reflexes were normal.

**Figure 1:**
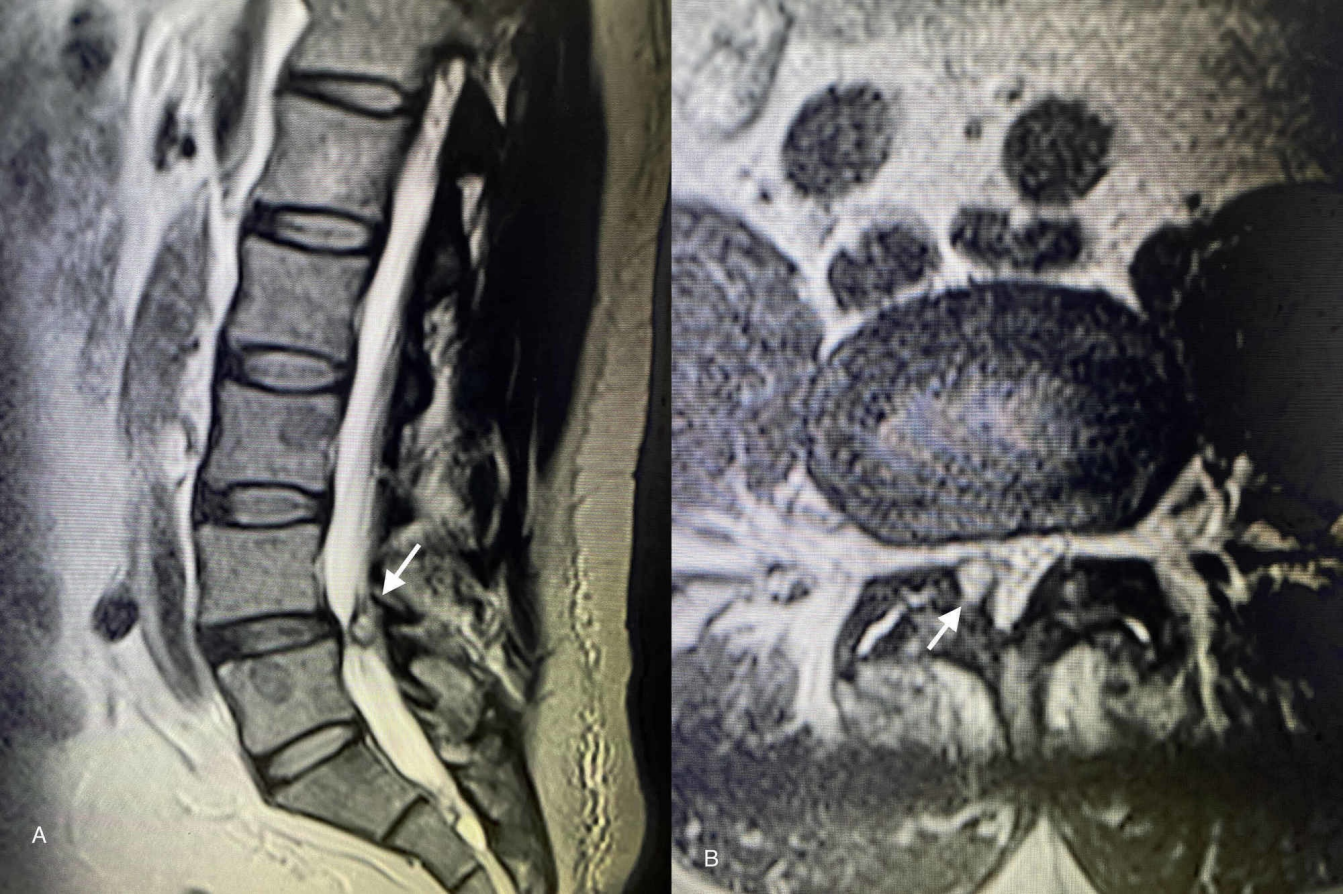
Magnetic resonance images of facet joint cysts (FJCs) at the L4-L5 vertebral level (A) Sagittal T2-weighted image demonstrating prominent FJC (white arrow) and grade 1 spondylolisthesis. (B) Axial T2-weighted image demonstrating the cyst (white arrow) arising from the right facet joint and obstructing the right lateral recess with compression of the thecal sac.

MRI demonstrated significant L4/L5 bilateral facet arthropathy along with grade 1 anterolisthesis. A well-defined synovial cyst was seen extending centrally from the right L4/L5 facet joint and obstructing the right lateral recess with compression of the thecal sac. Severe stenosis of the spinal canal was seen (Figure 1).

**Figure 2:**
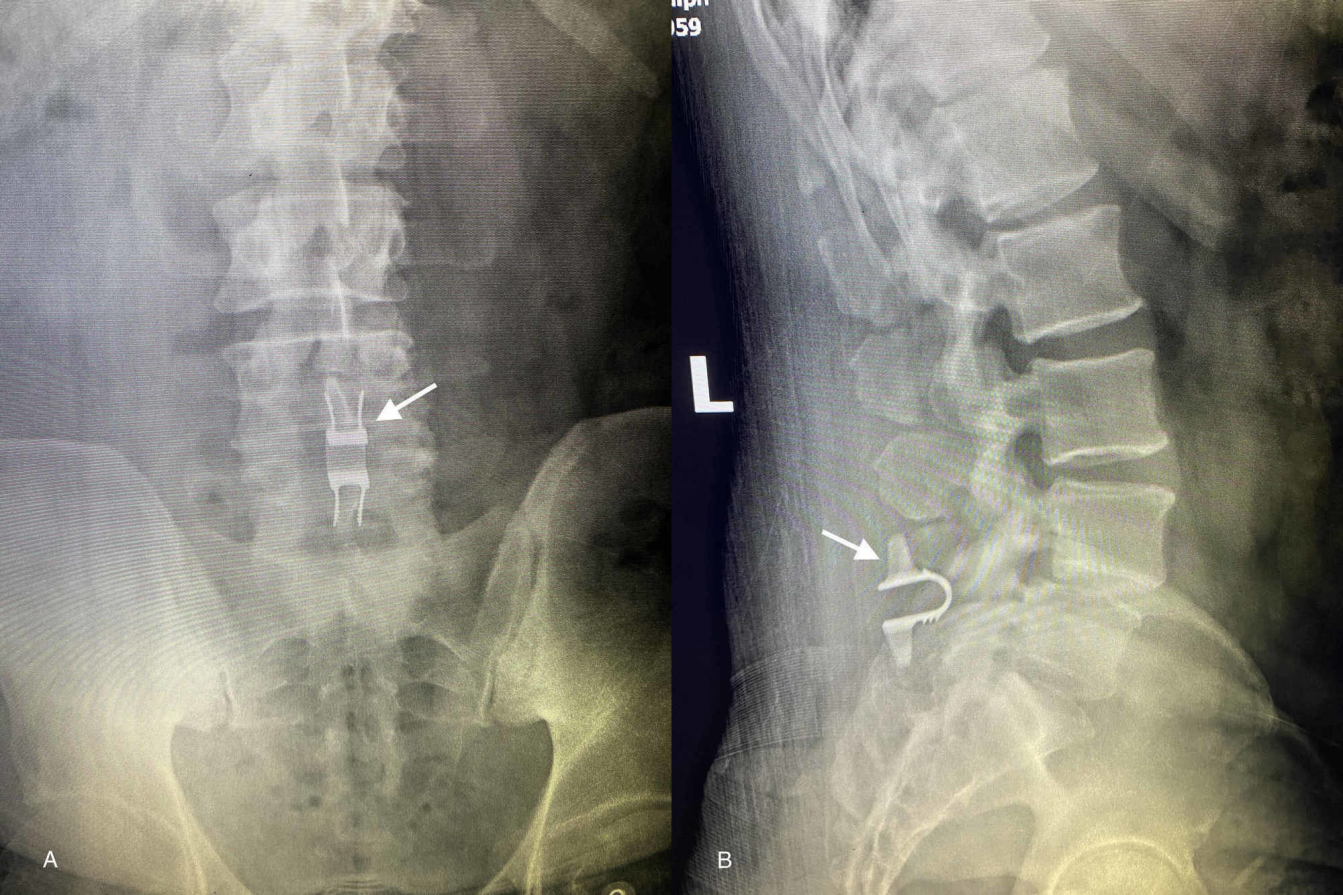
Two week postoperative Coflex® ILS implant radiographs Anteroposterior (A) and lateral (B) radiographs obtained two weeks postoperatively showing Coflex ILS implant (white arrows) in appropriate position at the L4-L5 level. ILS: Interlaminar stabilization

Decompression of the thecal sac at L4/5 was accomplished via bilateral laminotomies using a standard posterior midline approach. The FJC was identified on the right and was completely excised. Decompression of the nerve roots was additionally attained via medial partial facetectomies taking care not to further the instability. Following adequate decompression, a Coflex ILS implant sized to 14 mm was placed between the L4/L5 spinous processes and remaining lamina. An FJC was confirmed on histological examination. Postoperatively, the patient awoke neurovascularly intact while reporting immediate resolution of pain and radiculopathy in his right leg. Recovery was uneventful, and he ambulated with physical therapy on postoperative day 2. Postoperative radiographs are shown in Figure 2.

The patient had routine follow-up at two weeks and one month. At his one-month postoperative visit, his incision was well healed without drainage or signs of infection, and the patient was satisfied with the outcome of the operation. At his six-month follow-up, the patient continues to be pain-free without symptoms of radiculopathy.

## Discussion

FJCs are an increasingly reported cause of lower back pain, radiculopathy, and neurological deficits due to their proximity to adjacent nerve roots [5,10]. Various clinical manifestations have been reported in the literature ranging from incidental findings observed on imaging to compression of the cauda equina causing severe neurological deficits [3,6]. The etiology of these cysts has not yet been fully elucidated, although they appear to be related to degenerative changes in the facet joints themselves [1]. According to one theory, as intervertebral discs degenerate with time, the load-bearing capacity of the anterior column is shifted towards the posterior column lending to an increased load on the facet joints [3]. Subsequent weakening of the facet joint capsule leads to formation of a cyst that may be filled with synovial fluid or gas [5,10,11].

Initial treatment of an FJC typically involves conservative measures, which include physical therapy, oral analgesics, braces, chiropractic care, and corticosteroid injections [5,7,12]. These measures rarely result in spontaneous regression of an FJC, often providing only short-term relief [5]. Thus, surgical approaches are often warranted especially as it pertains to patients with progressive radiculopathy or neurological deficits that are refractory to non-operative management. Current management recommendations for FJCs include cyst excision accompanied by removal of a posterior portion of the vertebrae in order to decompress the thecal sac with or without an instrumented fusion depending on any iatrogenic instability caused by the partial or complete facetectomy [4,5,7].

However, with such a rigid fusion, there are particular considerations that need to be addressed. Although instrumented posterolateral fusion is an appropriate treatment for a grossly unstable spine, such fusion can be considered an extensive measure in treating a spine with little significant measurable instability, as is the case in our patient. In addition, long-term side effects related to unfused adjacent segment disease and instrumentation-related complications are important factors to consider [8].

The Food and Drug Administration (FDA)-approved Coflex ILS implant is a novel titanium, U-shaped device designed to be inserted into the interlaminar space and has proven to be a viable alternative to instrumented fusion [8]. Acting as a dynamic, motion-preserving stabilizer, the device functions essentially as a third joint, mechanically offloading the two facet joints while maintaining normal spinal dynamics. As demonstrated in recent studies, decompression plus Coflex ILS implantation has been shown to provide significant functional improvement with clear benefits of minimal blood loss, shorter length of hospital stay, and decreased morbidity [8,13-15].

Schmidt et al. recently conducted a prospective trial and randomized patients to either decompression alone (DA) or decompression and interlaminar stabilization (D + ILS) [13]. Composite clinical success (CCS) was defined as a term combining a number of key outcomes: functional improvement via Oswestry Disability Index (ODI) scores, re-operations or revision surgeries, the presence of new or worsening neurological deficits, and device-related complications [13]. CCS was calculated as statistically superior for patients treated with D + ILS; Moreover, an increase in walking tolerance and a decrease in compensatory pain management were noted in these patients. A recent study by Bae et al. also compared decompression and ILS to decompression and instrumented spinal fusion; CCS rates for patients treated with ILS were shown to be statistically superior, demonstrating the benefits of ILS in terms of durability [8]. However, regarding complications associated with ILS, similar rates were seen in both decompression with fusion and decompression with ILS [15]. Randomized prospective trials with a longer follow-up duration are needed to evaluate outcomes and device-related complications.

## Conclusions

Here, we discuss the case of a patient with an FJC causing severe right leg radiculopathy and neurological deficits. Surgical excision, decompression, and implantation of a Coflex ILS implant resulted in immediate improvement of his symptoms. This case highlights the management of an FJC and demonstrates a novel treatment alternative. Compared to a rigid instrumented spinal fusion, our treatment plan allowed for a faster recovery, shorter operative time, and minimal blood loss. As outpatient minimally invasive spine surgery becomes part of our new normal, this procedure can become a valuable part of our armamentarium.
